# Icariin attenuates neuroinflammation and exerts dopamine neuroprotection via an Nrf2-dependent manner

**DOI:** 10.1186/s12974-019-1472-x

**Published:** 2019-04-22

**Authors:** Bei Zhang, Guoqing Wang, Jingyi He, Qiuyu Yang, Daidi Li, Jingjie Li, Feng Zhang

**Affiliations:** Key Laboratory of Basic Pharmacology of Ministry of Education and Joint International Research Laboratory of Ethnomedicine of Ministry of Education, Zunyi Medical University, Zunyi, Guizhou China

**Keywords:** Parkinson’s disease, Neuroinflammation, Nrf2, Icariin, Neuroprotection

## Abstract

**Background:**

Oxidative stress and neuroinflammation are considered the major central events in the process of Parkinson’s disease (PD). Nrf2 is a key regulator of endogenous defense systems. New finds have contacted activation of Nrf2 signaling with anti-inflammatory activities. Therefore, the outstanding inhibition of neuroinflammation or potent Nrf2 signaling activation holds a promising strategy for PD treatment. Icariin (ICA), a natural compound derived from *Herba Epimedii*, presents a number of pharmacological properties, including anti-oxidation, anti-aging and anti-inflammatory actions. Recent studies have confirmed ICA exerted neuroprotection against neurodegenerative disorders. However, the underlying mechanisms were not fully elucidated.

**Methods:**

In the present study, mouse nigral stereotaxic injection of 6-hydroxydopamine (6-OHDA)-induced PD model was performed to investigate ICA-conferred dopamine (DA) neuroprotection. In addition, adult Nrf2 knockout mice and primary rat midbrain neuron-glia co-culture was applied to elucidate whether ICA-exerted neuroprotection was through an Nrf2-dependent mechanism.

**Results:**

Results indicated that ICA attenuated 6-OHDA-induced DA neurotoxicity and glial cells-mediated neuroinflammatory response. Furtherly, activation of Nrf2 signaling pathway in glial cells participated in ICA-produced neuroprotection, as revealed by the following observations. First, ICA enhanced Nrf2 signaling activation in 6-OHDA-induced mouse PD model. Second, ICA failed to generate DA neuroprotection and suppress glial cells-mediated pro-inflammatory factors production in Nrf2 knockout mice. Third, ICA exhibited neuroprotection in primary neuron-glia co-cultures but not in neuron-enriched cultures (without glial cells presence). Either, ICA-mediated neuroprotection was not discerned after Nrf2 siRNA treatment in neuron-glia co-cultures.

**Conclusions:**

Our findings identify that ICA attenuated glial cells-mediated neuroinflammation and evoked DA neuroprotection via an Nrf2-dependent manner.

**Electronic supplementary material:**

The online version of this article (10.1186/s12974-019-1472-x) contains supplementary material, which is available to authorized users.

## Highlights

- Oxidative stress and neuroinflammation are considered as the major central events in the process of Parkinson’s disease
- Activation of Nrf2 signaling has been linked to anti-inflammatory activities
- Icariin attenuates glial cells-mediated neuroinflammation and evoked dopamine neuroprotection via an Nrf2-dependent manner

## Introduction

Parkinson’s disease (PD) is a multifactorial neurodegenerative disease accompanied by progressive loss of dopamine (DA) neurons in the midbrain substantia nigra (SN) [1]. The clinical manifestation of PD mainly includes dyskinesia, resting tremor, muscle rigidity, and gait disturbance [2]. Currently, there is no effective treatment for PD.

Although the cause of PD is not completely clear, there is an age-related excessive oxidative stress, leading to neuroinflammation, DA auto-oxidation, a-synuclein accumulation, and glial cells activation [3, 4]. Nuclear factor erythroid-2 related factor 2 (Nrf2), a key regulator of redox homeostasis, controls acute/chronic inflammation [5, 6]. Nrf2 activation initiates phase II enzymes expressions, such as heme oxygenase-1 (HO-1) and NADPH quinone oxidoreductase 1 (NQO1), which mitigates the pathogenesis of neurodegenerative diseases [7]. However, insufficient Nrf2 activation has been highly involved in the progress of neurodegenerative diseases. In addition, the anti-inflammatory properties of Nrf2 are well established. Amounts of evidence presented a transcriptional repression of pro-inflammatory cytokines, including tumor necrosis factor-α (TNF-α), interleukin-1β (IL-1β), and nitric oxide (NO), in microglia and astroglia following Nrf2 activation [1, 8]. Moreover, a Nrf2 activator, dimethyl fumarate (DMF)-mediated DA neuroprotection has been involved in inhibiting activation of microglia and astroglia [9]. Also, Nrf2-mediated neuroinflammation has been considered to the primary therapeutic targets for amyotrophic lateral sclerosis (ALS) [10, 11]. Together, the outstanding inhibition of neuroinflammation or potent Nrf2 signaling activation holds a promising strategy for neurodegenerative diseases treatment.

Icariin (ICA) is a flavone compound extracted from *Herba Epimedii* and shows a range of pharmacological effects, such as anti-oxidant, anti-inflammation, and anti-aging actions [12, 13]. Furtherly, ICA could pass the blood-brain barrier and alleviates inflammatory infiltration in rat models of neurological disorders. For example, ICA attenuated lipopolysaccharide (LPS)-induced learning and memory deficits in rats by inhibiting the production of pro-inflammatory factors in hippocampus [14]. In the PD animal model, ICA protected DA neuronal damage against LPS-induced neurotoxicity [15]. However, the specific target and the underlying mechanisms underlying ICA-elicited neuroprotection are not fully illuminated.

In the present study, we detected the neuroprotective effects of ICA against 6-hydroxydopamine (6-OHDA)-induced DA neurotoxicity both in vivo and in vitro. Furthermore, adult Nrf2 knockout mice and primary rat midbrain neuron-glia co-culture was validated to elucidate whether ICA-exerted DA neuroprotection was via an Nrf2-dependent mechanism.

## Materials and methods

### Reagents

ICA (purity > 98%) was purchased from Nanjing Zelang Biological Technology Co., Ltd. (Nanjing, China). 6-OHDA was obtained from Sigma-Aldrich (St. Louis, MO). Sytox green nucleic acid fluorescence stain was bought from Bio-Rad (Hercules, CA, USA). Anti-tyrosine hydroxylase (TH), HO-1, NQO1, glial cell line-derived neurotrophic factor (GDNF) and brain-derived neurotrophic factor (BDNF) antibodies were obtained from Abcam (Cambridge, MA, USA). Anti-Kelch-like ECH-associated protein 1 (Keap1), Nrf2, proliferating cell nuclear antigen (PCNA), ionized calcium-binding adapter molecule-1 (Iba-1), glia fibrillary acidic protein (GFAP), inducible nitric oxide synthase (iNOS), TNF-α, and β-actin antibodies were bought from Proteintech Group (Chicago, IL, USA). Nrf2-siRNA and control-siRNA were purchased from Thermo Fisher Scientific (Waltham, MA, USA).

### Animals and treatment

Male wild type (WT) mice and male homozygous Nrf2 knockout (Nrf2 KO) mice (25–30 g, 8–10 weeks) were purchased from the Model Animal Research Centre of Nanjing University (Nanjing, China). All experimental procedures were carried out in accordance with Chinese Guidelines of Animal Care and Welfare and this study received an approval from the Animal Care and Use Committee of Zunyi Medical University (Zunyi, China). WT and Nrf2 KO mice received intragastric administration with ICA (60 mg/kg) once daily for 10 consecutive days. On the third day, mice were injected stereotactically with 6-OHDA (4 μg, in 0.2% ascorbic acid) in the SN on the left side of the brain with coordinates from Bregma: AP − 2.2 mm, ML 1.4 mm, DV − 4.7 mm [16]. Normal control animals accepted equal volume saline.

### Rotarod test

The rotarod test was widely used to detect the muscular coordination and balance [17]. Prior the test start, all mice were trained to stay at 0 rpm for a while, then steadily increased to 10 rpm in 30 s and 5 rpm per 30 s until the mice slid off the steps. Animal behavioral activity was detected for 3 repeated trials on 1 day, and the average duration of stay on the rod was recorded.

### Open field test

Open field test was performed at the last ICA application for evaluating the levels of anxiety emotionality of animals [18]. Mice were placed on the open field and each mouse was placed in a separate area and its behavioral parameters were recorded during the 5 min. The device was washed with 75% alcohol solution before each next behavioral test in order to eliminate the odors from the previous mouse. The distance of mice in central and peripheral activities was recorded by computer. After the experiment, the total distance of mouse movement was counted.

### Ultra-high-performance-liquid chromatography (UHPLC) analysis

Mouse striatum levels of DA, DOPAC, and HVA were detected by Agilent 1290 Infinity II series UHPLC System (Agilent Technologies). Striatum tissues were sonicated in extract solvent (acetonitrile-methanol-water, 2:2:1, 2% formic acid). The homogenate was centrifuged, and a 100 μl aliquot of the clear supernatant was transferred to an auto-sampler vial for UHPLC-MS/MS analysis. The mobile phase A contained 10 mmol/L ammonium acetate/0.1% formic acid, and the mobile phase B was acetonitrile.

### Immunofluorescence staining

Thirty-five-micrometer-thick brain sections stained with the corresponding antibodies. Briefly, brain slices were incubated with 0.3% Triton X-100 and closed with goat serum. Subsequently, brain slices concentrated with anti-TH (1:500), Iba-1 (1:200), and GFAP (1:300) antibodies at 4 °C overnight, respectively. Then, the slices were incubated with goat anti-rabbit antibody (green) secondary antibodies (1:1500) for 1 h. Digital images of TH-positive neurons, Iba-1-positive microglia, and GFAP-positive astroglia were obtained via Olympus microscope (Olympus, Tokyo, Japan). DA damage was assessed through the quantification of TH-positive neurons.

### Western blotting

Nuclear and cytosol fractions were extracted from the mice midbrain tissues with Nuclear-Cytosol Extraction Kit (Solarbio, Beijing, China) following the manufacture’s protocols. Total proteins were isolated by RIPA lysis solution (Solarbio, Beijing, China). Protein levels were quantified using BCA assay. Equal amounts of protein (10–30 μg/lane) were separated on 10% Bis-Tris Nu-PAGE gel. Then, the membranes were incubated with primary antibodies: TH (1:2000), Iba-1 (1:800), GFAP (1:1000), Nrf2 (1:1500), Keap1 (1:2000), HO-1 (1:10000), NQO1 (1:2000), PCNA (1:2000), TNF-α (1:800), iNOS (1:1500), GDNF (1:800), BDNF (1:1000), and β-actin (1:4000). Next day, the membranes were incubated with anti-rabbit IgG secondary antibodies at 1:5000 for 1 h. Finally, the blots were detected using ECL substrate. β-actin was used as an internal standard to monitor loading errors. Densitometric analysis of immunoblots was performed using the Quantity One (Bio-Rad, Hercules, CA, USA) software system. The ratio of densitometry values of purpose protein with β-actin was analyzed and normalized to each respective control groups.

### Real-time RT-PCR assay

The total RNA of mouse midbrain tissues was prepared using RNeasy kit and the detailed steps of Real-time RT-PCR were described previously [19]. Nrf2, Keap1, HO-1, NQO1, and β-actin genes were tested. Accordingly, the genes’ expression was normalized with β-actin. The primer sequences were as follows.

**Table:** 

Gene	Source	Forward primer (5′-3′)	Reverse primer (5′-3′)
Nrf2	Mouse	CAGTCTTCACCACCCCTGAT	CAGTGAGGGGATCGATGAGT
Keap1	Mouse	AGGAATGAGTGGCGGATGAT	GCGCTCCACACTGTTCAACT
HO-1	Mouse	AGAGGCTAAGACCGCCTTCC	TCTGACGAAGTGACGCCATC
NQO1	Mouse	AGCCAATCAGCGTTCGGTAT	AGCCAATCAGCGTTCGGTAT
β-actin	Mouse	GTGCTATGTTGCTCTAGACTTCG	ATGCCACAGGATTCCATACC

### Primary rat midbrain neuron-glia and neuron-enriched cultures

Female pregnant SD rats (250-300 g) were purchased from the Experimental Animal Center of the Third Military Medical University (Chongqing, China). Primary midbrain neuron-glia cultures were prepared from the ventral mesencephalic tissues of embryonic rats 14–15 days age [20]. Then, the rat whole brains were dissected and the midbrain were isolated. After excision of blood vessels and meninges, the midbrain tissues were mechanically triturated and the dissociated cells were planted in poly-d-lysine-coated 24-well plates or 96-well plates. Seven-day-old cultures were employed for drug treatment. During treatment, immunocytochemical staining demonstrated that primary neuron-glia co-cultures composed of 50% astrocytes, 10% microglia, 40% neurons, and 1% DA neurons. In addition, midbrain neuron-enriched cultures were prepared by adding cytosine β-D-arabinofuranoside (8 μM) in primary neuron-glia cultures to inhibit glial cells proliferation [21]. ICA treatment was performed with 7 days of cultures followed by 6-OHDA (40 μM) intervention [15].

### Primary rat mixed-glia cell cultures

Primary rat mixed-glia cultures were prepared from the whole brains of 1-day-old rat pups [22]. The meninges and blood vessels were isolated, the brain tissues were homogenized and the cells (2 × 10^6^/well) were planted in a poly-d-lysine-coated 6-well plate. Seven-day-old mixed-glial cells were cultured for drug treatment.

### Nrf2-siRNA transfection

Primary mixed-glia cells were transfected with Nrf2-siRNA (40 nmol/L) or control-siRNA (40 nmol/L) for 24 h. Western blotting and real-time RT-PCR were used to test the transfection rate.

### Neuron-glia reconstituted cultures using transwell

First, primary neuron-enriched cultures were planted in 24-well culture plates. Mixed-glia cultures were planted in the transwell. Seven days later, the mixed glial cells in transwell were processed by Nrf2-siRNA for 1 day. Then, the mixed glial cells were transferred to 7-day neuron-enriched cultures within fresh medium. Accordingly, the reconstituted neuron-glia cultures were treated with ICA and 6-OHDA for 7 days. DA neurons damage was assessed by DA neuronal quantification and TH protein expression detection.

### Immunocytochemical staining

Cells were fixed with 4% paraformaldehyde followed by permeabilization using Triton X-100 and closed with goat serum. DA neurons were labeled with anti-TH (1:500) antibody at 4 °C overnight and then incubated with anti-rabbit-IgG (1:1500) antibody for 1 h. TH-positive neurons numbers were calculated from three wells, and three randomly selected areas were analyzed for each group.

### Statistical analysis

All data were presented as mean ± SEM. Statistical comparisons were analyzed using GraphPad Prism 5 (GraphPad Software Inc., La Jolla, CA, USA) by one-way ANOVA. After ANOVA expressed significant differences, the Bonferroni’s post hoc *test* was used for all pairwise comparisons among means. Statistical significance was considered as *p* < 0.05.

## Results

### ICA ameliorated 6-OHDA-induced DA neuronal damage

**Fig. 1:**
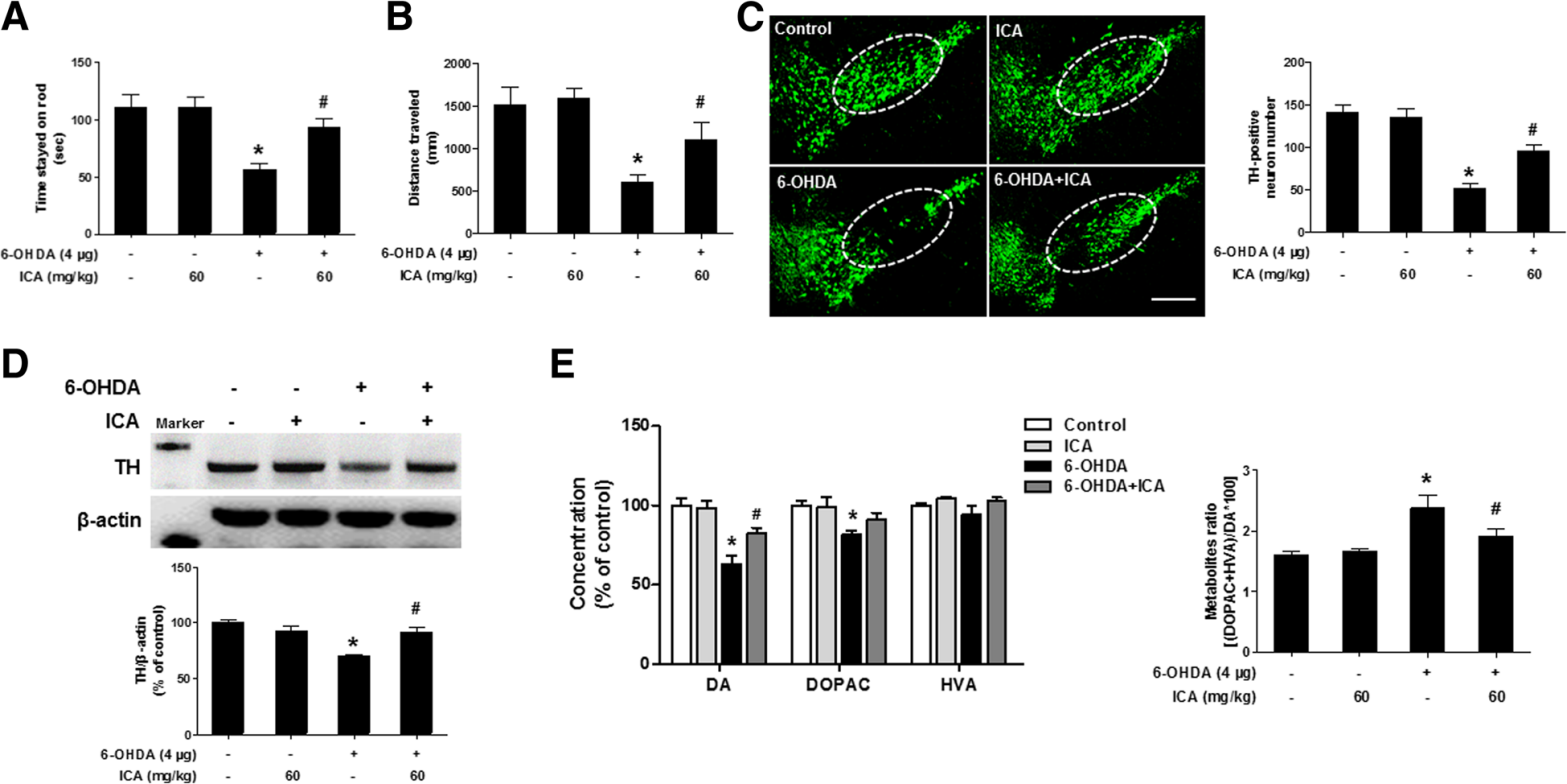
ICA ameliorated 6-OHDA-caused DA neuronal damage. Mice were intragastrically given ICA (60 mg/kg) for 10 consecutive days and a single intranigral injection of 6-OHDA (4 μg) in the left side of SN beginning 3 days after ICA treatment. After the last treatment of ICA, rotarod test (**a**) and open-field test (**b**) were performed. TH-positive neurons were measured by immunofluorescence staining (**c**). TH protein expression in midbrain was tested by western blot assay (**d**). The levels of DA, DOPAC and HVA in striatal tissues were detected by UHPLC. Meanwhile, the DA metabolite ratio [(DOPAC + HVA)/DA × 100] was computed (**e**). Results were mean ± SEM from 5 to 6 mice. *p < 0.05 compared with the control group, ^#^*p* < 0.05 compared with 6-OHDA-treated group. Scale bar = 200 μm

Neuroprotective effects of ICA on 6-OHDA-induced DA neuronal damage were investigated in WT mice. We first examined animal behavior changes via the rotarod and open-field tests. As shown in Fig. 1a and b, 6-OHDA reduced the time mice stayed on the rod and locomotor distance compared with control group. However, ICA attenuated 6-OHDA-induced decrease on these 2 tests. Furthermore, in both control and ICA alone group, strong TH immunoreactivity in midbrain SN was detected, whereas the TH immunoreactivity was interrupted in 6-OHDA group. ICA pretreatment protected DA neurons from 6-OHDA-induced neurotoxicity as shown by the enhanced TH-positive neurons number (Fig. 1c). The further quantification of DA neurons confirmed similar observations (Fig. 1d). As illustrated in Fig. 1e, compared with control group, 6-OHDA reduced striatum DA and DOPAC levels and had no significant effects on HVA content. Compared with 6-OHDA group, ICA increased DA concentration and did not attenuate the levels of HVA and DOPAC. Moreover, the DA metabolite ratio was increased in the 6-OHDA group and ICA treatment turned over this ratio (Additional file 1).

### ICA activated Nrf2 signaling pathway

**Fig. 2:**
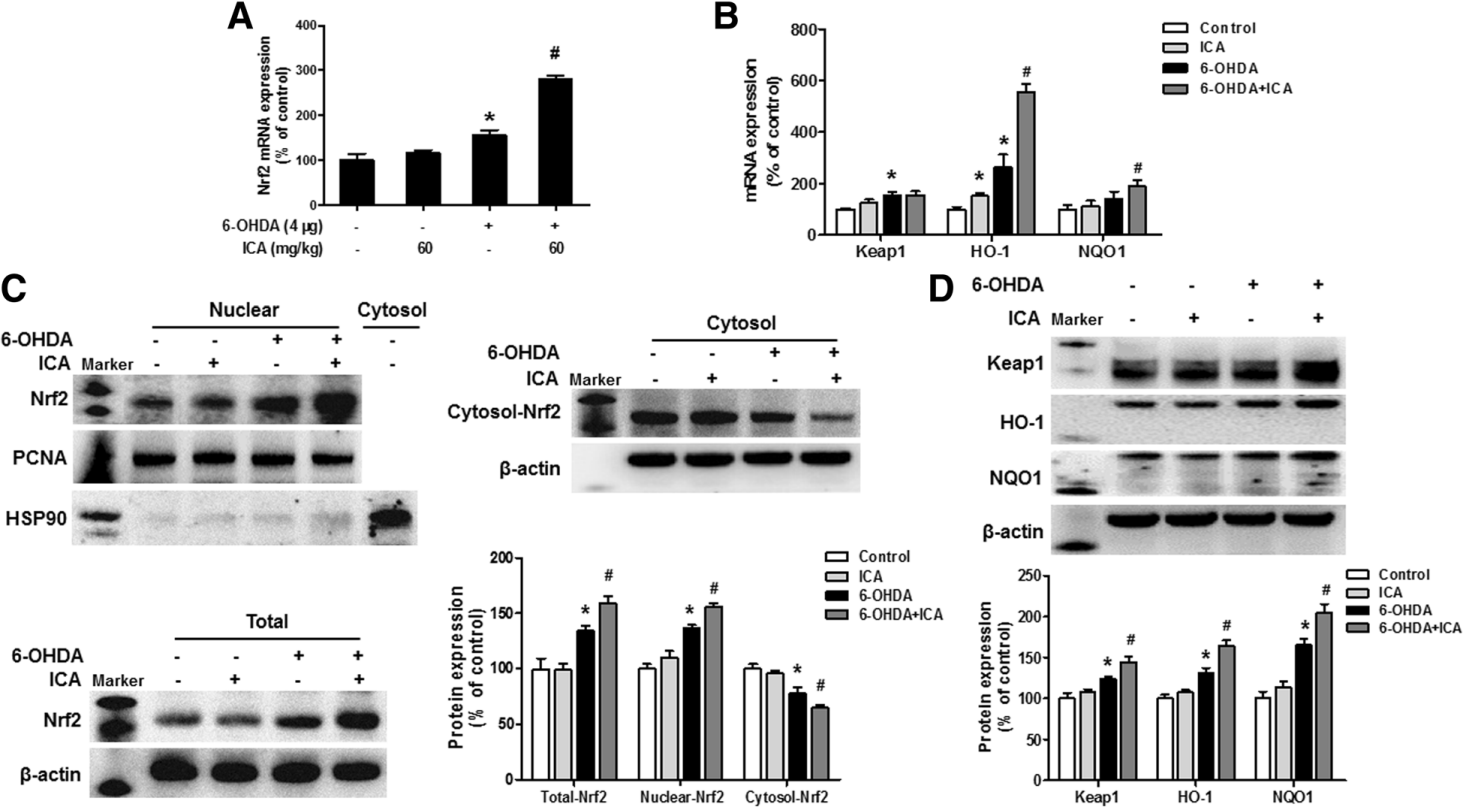
ICA activated Nrf2 signaling pathway. After ICA treatment for 10 days, the mRNA levels of Nrf2, Keap1, HO-1, and NQO1 in midbrain SN were detected by real-time RT-PCR (**a** and **b**). The protein expressions of nuclear Nrf2, cytosol Nrf2, HSP90, total Nrf2, Keap1, HO-1, and NQO1 in midbrain were detected via western blotting (**c** and **d**). Results were mean ± SEM from 5 to 6 mice. *p < 0.05 compared with the control group, ^#^*p* < 0.05 compared with 6-OHDA-treated group

To confirm whether ICA could activate Nrf2 signaling pathway in mice midbrain SN, the expression of Nrf2, Keap1, HO-1, and NQO1 were detected by RT-PCR and western blot assay. As shown in Fig. 2a, Nrf2 mRNA expression was upregulated in 6-OHDA and 6-OHDA+ICA treatment group. Meanwhile, Keap1, HO1, and NQO1 mRNA levels were more prominent after 6-OHDA alone or co-treatment with ICA exposure (Fig. 2b). Additionally, ICA increased the total protein expression of Nrf2. Furthermore, to further determine whether ICA could affect Nrf2 distribution, the cytosolic components and nuclear fractions were measured. We used the cytosolic marker HSP90 to verify the purity of nuclear proteins, the nuclear fraction was not contaminated by cytosolic proteins as confirmed by the absence HSP90. Next, western blot analysis showed that 6-OHDA and 6-OHDA combined with ICA increased the translocation of Nrf2 from the cytosol to the nuclear (Fig. 2c). Also, the higher protein expressions of Keap1, HO-1, and NQO1 were indicated in 6-OHDA and 6-OHDA+ICA groups than those in control and ICA alone groups (Fig. 2d).

### Nrf2 signaling participated in ICA-mediated neuroprotection

**Fig. 3:**
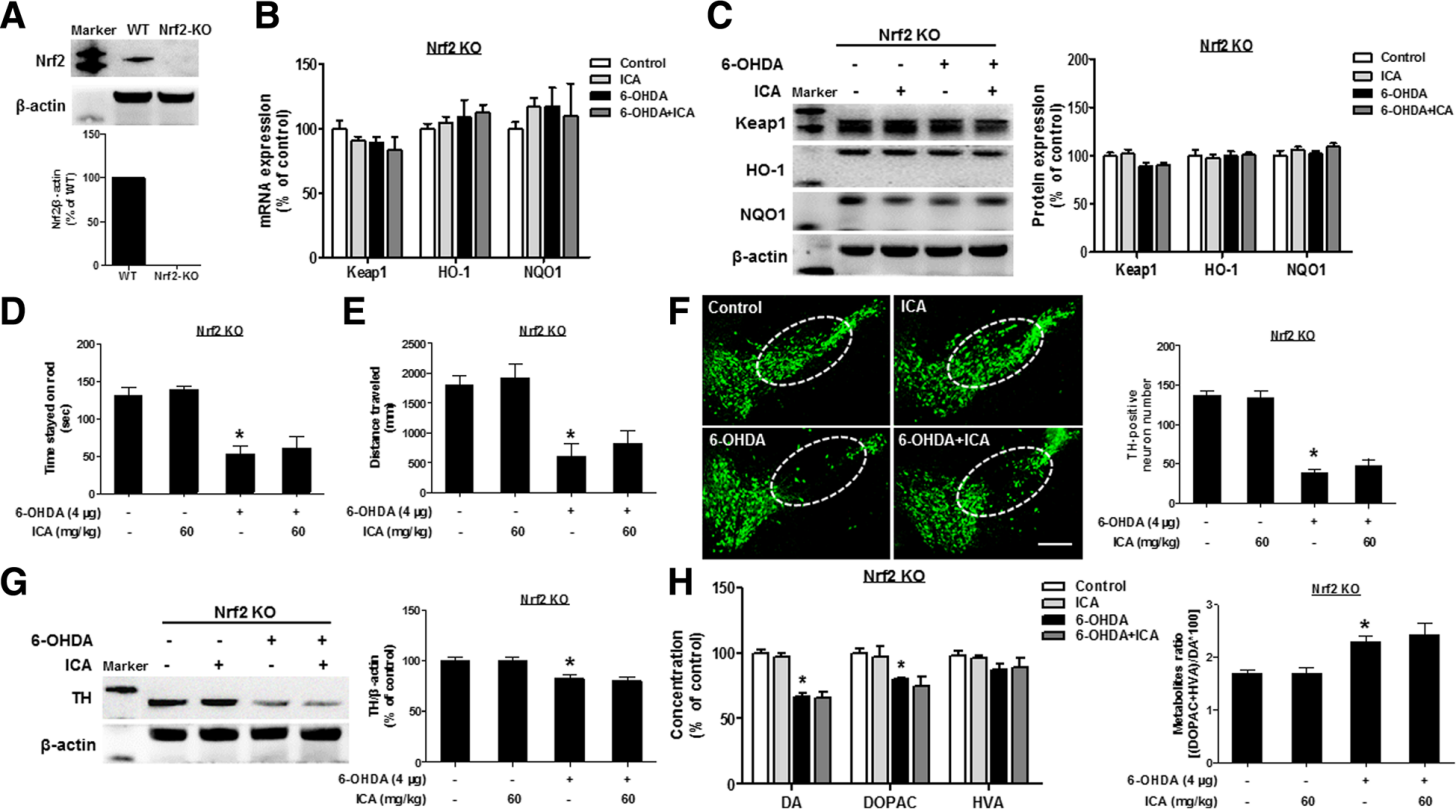
Nrf2 signaling participated in ICA-mediated neuroprotection. Nrf2 KO mice were treated with ICA (60 mg/kg) daily for 10 consecutive days and a single intranigral injection of 6-OHDA (4 μg) in the left side of SN 3 days after ICA treatment. Then, mouse brains were harvested and the knockout efficiency was tested by western blotting (**a**). The mRNA and protein expressions of Keap1, HO-1, and NQO1 in midbrain were detected by real-time RT-PCR (**b**) and western blotting (**c**), respectively. The rotarod test and open-field test were performed (**d, e**). TH-positive neurons were measured by immunofluorescence staining (**f**). TH protein expression in mesencephalon was detected by western blot assay (**g**). The levels of DA, DOPAC, and HVA in striatal tissue were tested by UHPLC. The DA metabolite ratio [(DOPAC + HVA)/DA × 100] was computed (**h**). Results were mean ± SEM from 5 to 6 mice. *p < 0.05 compared with the control group. Scale bar = 200 μm

Since ICA promoted Nrf2 distribution, Nrf2 KO mice were further conducted to evaluate the role of Nrf2 on ICA-mediated neuroprotection. Firstly, the Nrf2 knockout efficiency was tested by western blotting (Fig. 3a). Next, the gene and protein expressions of Keap1, HO-1, and NQO1 in midbrain were not altered in Nrf2 KO mice (Fig. 3b and c). Furthermore, ICA-ameliorated 6-OHDA-induced decrease of time stayed on stick and locomotor distance was not discerned in Nrf2 KO mice (Fig. 3d and e). Consistently, ICA-mediated DA neuroprotection was absent in Nrf2 KO mice via TH immunostaining and protein level detection (Fig. 3f and g).

Interestingly, as shown in Fig. 1c, 6-OHDA injection caused 62% DA neuron loss (54.0 ± 4.8 TH-positive neurons) in WT mice. While in Fig. 3f, 6-OHDA injection enhanced the neuronal loss into 71% (38.6 ± 3.4 TH-positive neurons) in Nrf2 KO mice. In addition, attenuation of DA metabolite level and ratio produced by ICA in Nrf2 KO mice appeared to be less evident than those in WT mice (Fig. 3h).

### ICA depressed glia cells-induced neuroinflammation through inhibiting Nrf2 signing activation

**Fig. 4:**
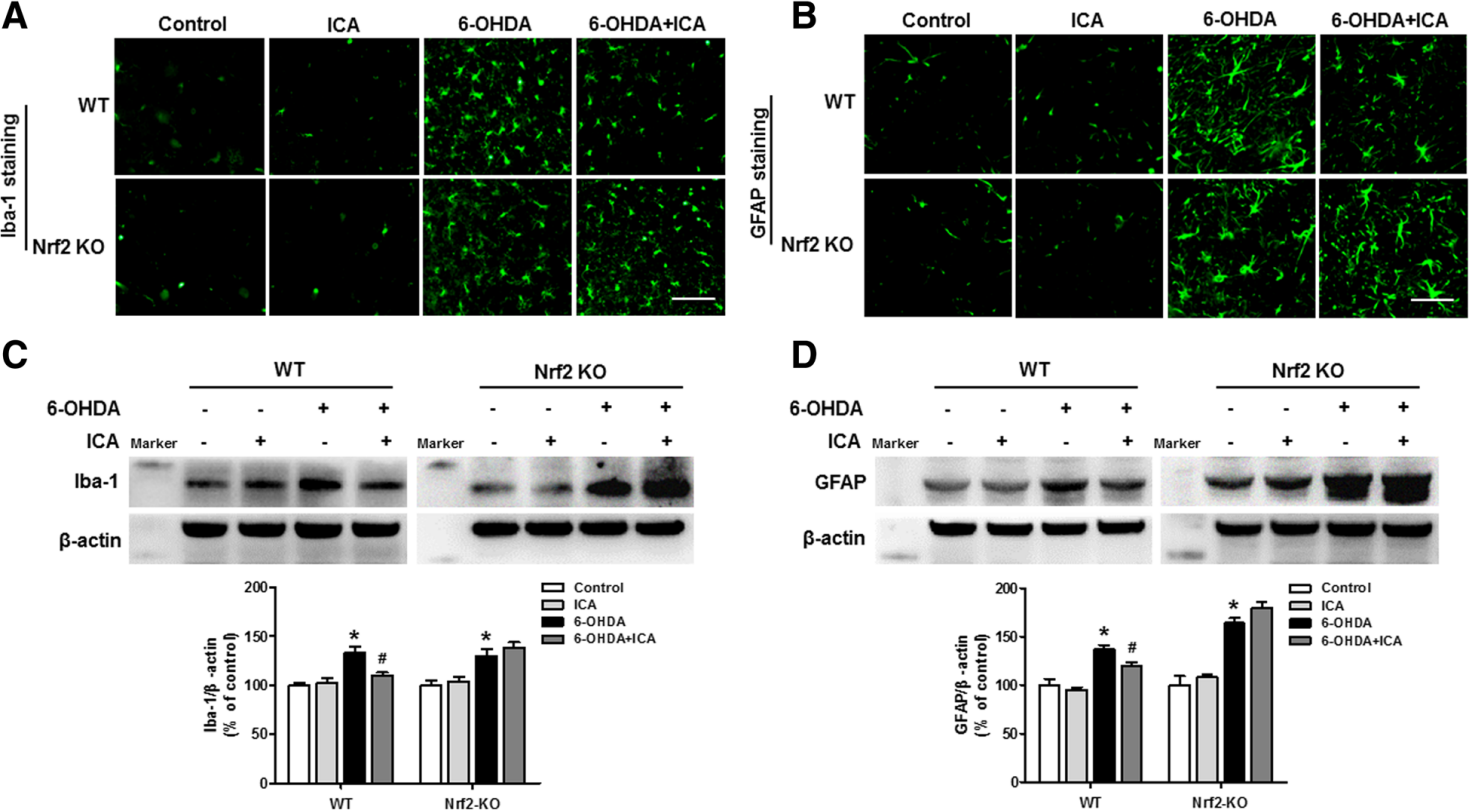
ICA depressed glial cells activation through activating Nrf2 signing. WT and Nrf2 KO mice were treated with ICA (60 mg/kg/d) with a single intranigral injection of 6-OHDA (4 μg) in the SN on the left side of the brain beginning 3 days after ICA treatment. Later, brain sections from WT and Nrf2 KO mice were immunostained with anti-Iba-1 (**a**) and GFAP (**c**) antibodies, respectively. The protein expressions of Iba-1 (**b**) and GFAP (**d**) in WT and Nrf2 KO mice were measured by western blotting. Results were mean ± SEM from 5 to 6 mice. *p < 0.05 compared with the control group, ^#^*p* < 0.05 compared with 6-OHDA-treated group. Scale bar = 100 μm

**Fig. 5:**
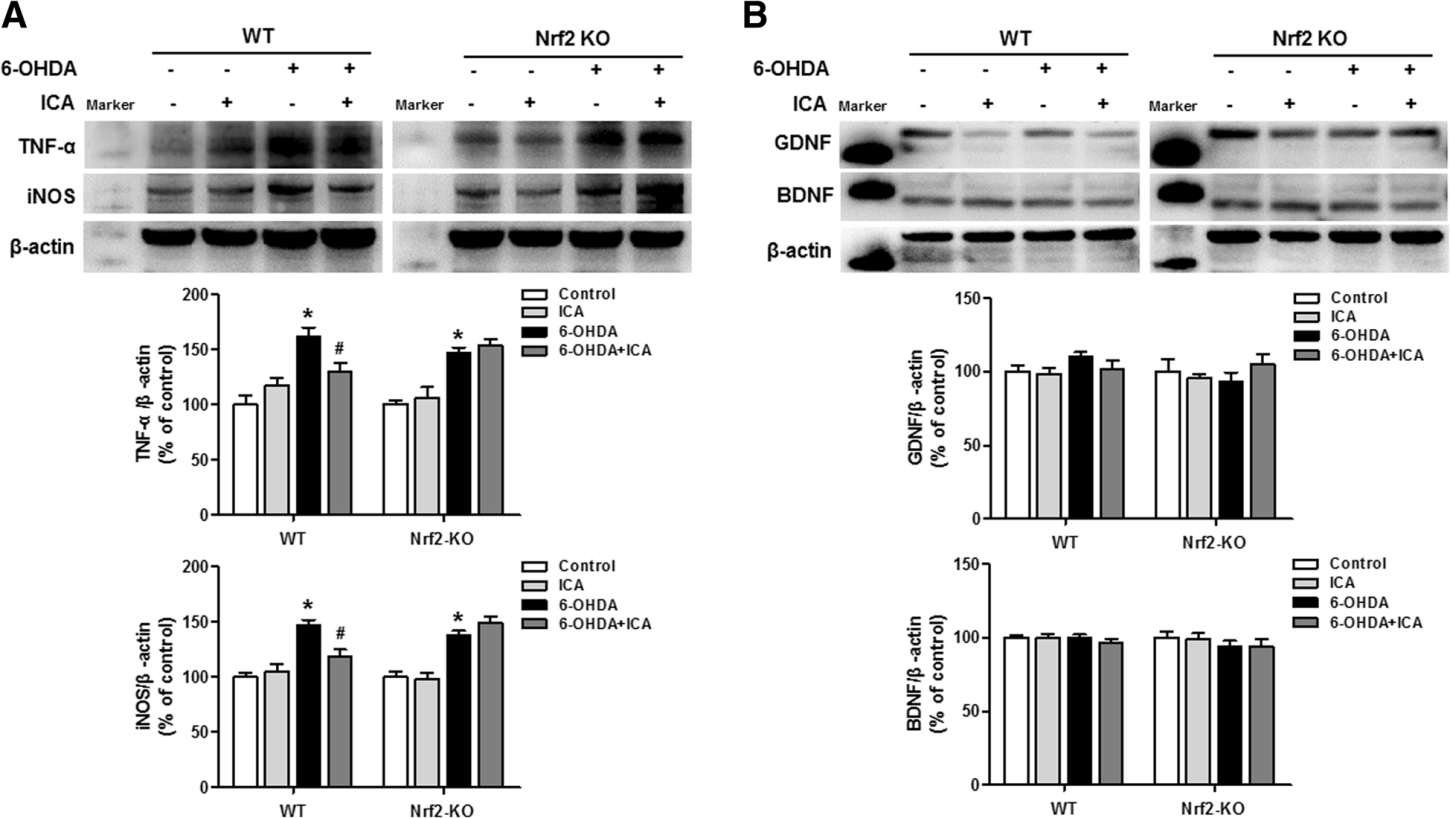
The role of Nrf2 signaling on ICA-affected functions of glial cells. After ICA treatment for 10 days, the protein levels of pro-inflammatory mediators, such as TNF-α and iNOS (**a**), and the production of neurotrophic factors, such as GDNF and BDNF (**b**), in the midbrain of WT and Nrf2 KO mice were measured by western blotting. Results were mean ± SEM from 5 to 6 mice. *p < 0.05 compared with the control group, ^#^*p* < 0.05 compared with 6-OHDA-treated group

It has been confirmed that glial cells-induced neuroinflammation participated in the process of DA neurodegeneration. Then, we detected the effects of ICA on neuroinflammation in 6-OHDA-caused DA neuronal damage. As indicated in Fig. 4a and c, ICA attenuated 6-OHDA-induced microglia and astroglia activation as evidenced by the less immunoreactivity of Iba-1 and GFAP shown in WT mice, whereas no obvious effects of ICA on the immunoreactivity in glial cells after 6-OHDA administration were discerned in Nrf2 KO mice. Meanwhile, Iba-1 and GFAP protein expression changes were parallel with the immunocytochemical analysis in WT and Nrf2 KO mice (Fig. 4b and d). Next, the actions of ICA on the function of glial cells were evaluated. As shown in Fig. 5a, ICA-induced initial decrease in TNF-α and iNOS protein expressions was observed compared with 6-OHDA group in WT mice. However, ICA failed to reduce TNF-α and iNOS protein levels after 6-OHDA stimulation in Nrf2 KO mice. On the other hand, both ICA and/or 6-OHDA had no significant effects on GDNF and BDNF production in WT mice and Nrf2 KO mice (Fig. 5b).

### ICA targeted glia cells Nrf2 to produce neuroprotection actions

**Fig. 6:**
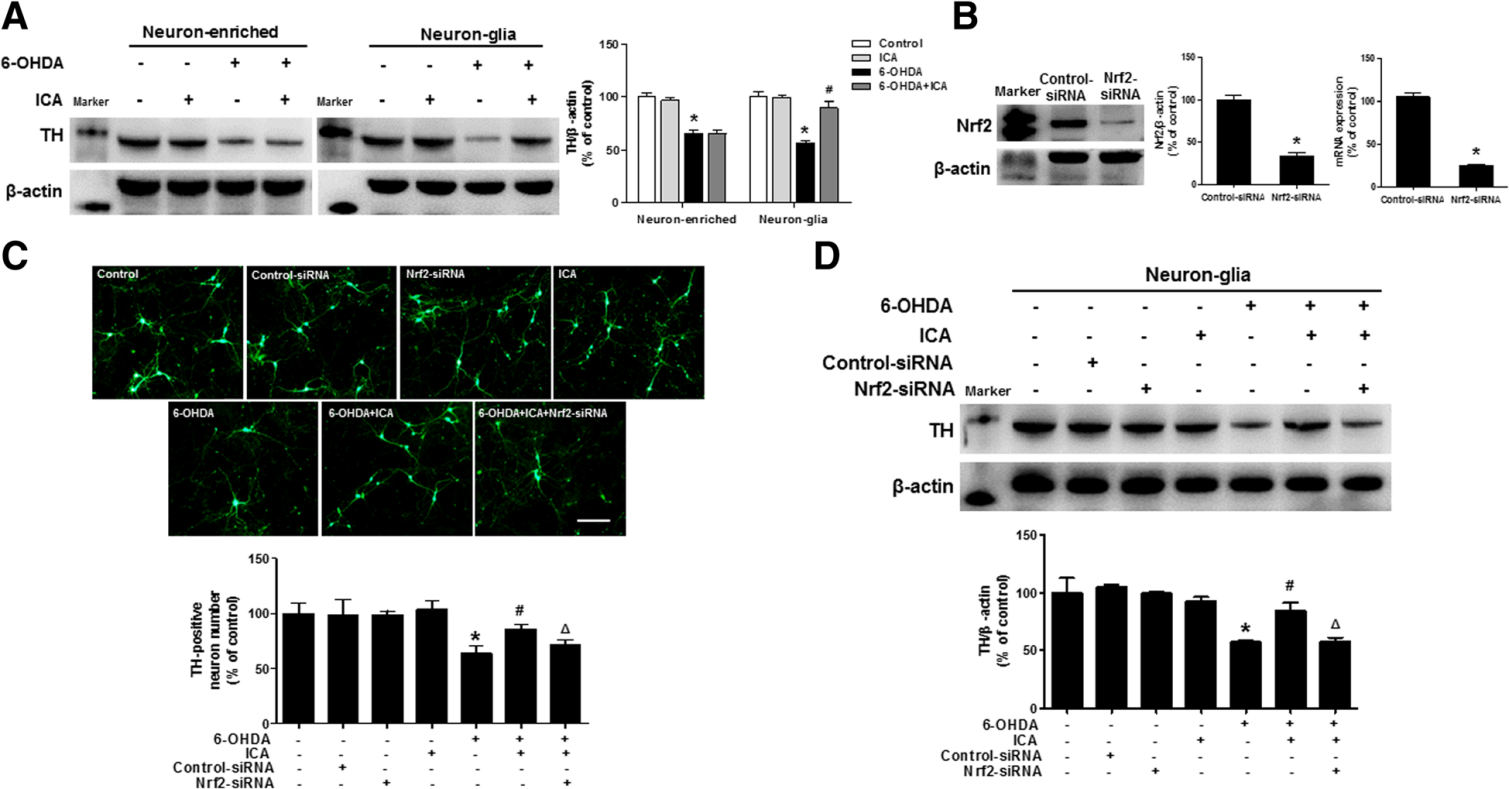
ICA targeted Nrf2 in glial cells to produce DA neuroprotection. Primary rat midbrain neuron-enriched and neuron-glia cultures were pretreated with ICA (0.1 μM) for 30 min followed by 6-OHDA (40 μM) stimulation for 7 days. TH protein expression was measured by western blot analysis (**a**). Primary mixed-glia in transwells were treated with Nrf2-siRNA (40 nM) for 24 h and the silence rate was verified by real-time RT-PCR and western blotting (**b**). Then, mixed-glial cells after silence were transferred to neuron-enriched cultures. After that the reconstituted neuron-glia co-cultures were treated with ICA and 6-OHDA for 7 days. DA neurons quantification was determined by immunocytochemical staining and western blotting with anti-TH antibody (**c** and **d**). Results were mean ± SEM from 3 independent experiments performed in triplicate. *p < 0.05 compared with the control cultures, ^#^*p* < 0.05 compared with 6-OHDA-treated cultures, ^△^*p* < 0.05 compared with 6-OHDA+ICA-treated cultures. Scale bar = 100 μm

To further explore which cell type ICA targeted to produce DA neuroprotection via the activation of Nrf2 signaling, primary rat midbrain neuron-glia cultures and neuron-enriched cultures were applied, treated with ICA for 30 min, and then stimulated by 6-OHDA. Seven days later, TH protein expression detection showed that ICA substantially attenuated 6-OHDA-induced DA neuronal injury in neuron-glia co-cultures, whereas no neuroprotection mediated by ICA was exhibited in neuron-enriched cultures, suggesting that glial cells were responsible for ICA-conferred DA neuroprotection (Fig. 6a). Subsequently, we examined whether ICA-mediated neuroprotection was attributable to the regulation of glial cells Nrf2 signaling. First, primary mixed-glial cells planted in transwell were processed with Nrf2-siRNA for 24 h. When Nrf2-siRNA was processed, the Nrf2 gene and protein levels were downregulated and the silence rate was approximately 75.5% (Fig. 6b). Then, Mixed-glial cells after silence were transferred to neuron-enriched cultures. Thus, the reconstituted neuron-glia cultures were established and treated with ICA and/or 6-OHDA for 7 days. DA neurons immunostaining and TH protein expression measurement indicated that there was no significant difference among the control, Nrf2-siRNA, control-siRNA, and ICA alone cultures. In addition, 6-OHDA-induced reduction in DA neuronal number and TH protein expression was ameliorated by ICA pretreatment. However, the addition of Nrf2-siRNA to glial cells neutralized ICA-protected DA neurons against 6-OHDA-attended neurotoxicity (Fig. 6c and d). Collectively, ICA activated Nrf2 signaling in glial cells to exert DA neuroprotection.

## Discussion

This study aimed at investigating the neuroprotective effects of ICA on 6-OHDA-induced DA neurotoxicity in vivo and the underlying mechanisms. Results demonstrated that ICA attenuated 6-OHDA-induced DA neurotoxicity and glia cells-mediated neuroinflammatory response. Furtherly, activation of Nrf2 signaling pathway in glial cells participated in ICA-produced neuroprotection, as revealed by the following observations. First, ICA enhanced Nrf2 signaling activation in 6-OHDA-induced mouse PD model. Second, ICA failed to generate DA neuroprotection and suppress glia cells-mediated pro-inflammatory factors production in Nrf2 knockout mice. Third, ICA exhibited neuroprotection in primary neuron-glia co-cultures but not in neuron-enriched cultures. Also, ICA-mediated DA neuroprotection was not discerned after Nrf2 siRNA treatment in neuron-glia co-cultures. Together, ICA inhibited glia cells-mediated neuroinflammation and afforded DA neuroprotection via the activation of Nrf2 signaling.

Maintainment of redox homeostasis in the CNS is dispensable for neuronal survival and development. Oxidative stress is generated by a continuous imbalance between the production and removal of reactive oxygen species (ROS) and reactive nitrogen species (RNS) [23]. The brain is recognized to be particularly sensitive to oxidative stress due to its high oxygen consumption and lipid level. In addition, oxidative stress serves as one of the major events participated in neuronal injury and loss in various chronic neurodegenerative diseases, such as Alzheimer’s disease (AD), Parkinson’s disease (PD), and Huntington’s disease (HD) [24]. The transcription factor Nrf2 is considered to be a master mediator of cellular homeostasis, since it controls the expressions of multiple cytoprotective genes [25]. The list of these cytoprotective genes is persistently growing to include those participated in biotransformation, metabolic reprogramming, and anti-oxidant defense. However, Nrf2 deficiency replicated transcriptomic changes in AD patients [26]. Also, Nrf2 inactivation associated with aging was the main risk factor for PD [27]. Moreover, the protective effects of Nrf2 signaling activation on ischemic injury was not indicated in Nrf2 knockout mice either [28]. Therefore, a functional Nrf2 signaling is identified as an important neuroprotective regulator of oxidative stress-related neurodegenerative disorders. In this study, ICA protected DA neurons against 6-OHDA-induced neurotoxicity, accompanied by the activation of Nrf2 signaling pathway. Furthermore, ICA failed to exert DA neuroprotection in Nrf2 knockout mice and primary rat midbrain neuron-glia co-culture treated by Nrf2 siRNA. Together, these results strongly suggested ICA-mediated DA neuroprotection might be attributable to the activation of Nrf2 signaling.

It is interesting to note that ICA targeted glia cells to exhibit neuroprotection as evidenced by this protection shown in primary neuron-glia co-cultures but not in neuron-enriched cultures. It is well known that neuroinflammation is an inevitable pathological process implicated in all types of neurological disorders [29]. The characteristic of neuroinflammation is glia activation, including microglia and astrocyte activation. Upon activation by brain damage, inflammation, and pathogens, glia cells present changes in their morphology and molecular repertoire. Actually, astrocyte became hypertrophied with an increased expression of GFAP [30], while microglia came to be hypertrophied and had the increased Iba-1 expression [31]. Following these morphological changes, microglia and astrocyte are referred to be reactive or activated. Most importantly, astrocytes and microglia release a battery of inflammatory cytokines and neurotoxic factors. The cumulation of these factors caused injury to the surrounding neuronal, particularly DA neuronal damage [32]. However, the continuous death of DA neurons, in turn lead to secondary activation of glia cells and the activated glia cells further secreted pro-inflammatory factors and thus caused DA neuronal loss [33]. Collectively, a vicious cycle to induce the prolonged neuroinflammation and the progressive reduction of DA neurons was created [34]. Therefore, attenuating activation of glia cells-elicited neuroinflammation might hold a promising strategy for the treatment of neuroinflammation-related disorders. In this study, ICA suppressed 6-OHDA-induced Iba-1 and GFAP activation and then reduced pro-inflammatory factors secretion. On the other hand, another result should be mentioned. Besides the involvement in the neuroinflammation, astroglia was the most abundant glial cells type in brain, participated in growth, nutrition, and repair of the nervous system [35]. Most importantly, astroglia are the major source of various neurotrophic factors, such as GDNF and BDNF. These neurotrophic factors have been confirmed to repair neurons and exert neuroprotection [36]. However, this study found that ICA inhibited 6-OHDA-induced GFAP activation but had no effect on BDNF and GDNF production, suggesting that astroglia-derived neurotrophic effects were not associated with ICA-mediated neuroprotection in this model.

Furthermore, new findings have contacted activation of Nrf2 signaling with anti-inflammatory activities. Activation of Nrf2 signaling could reduce the overproduction of pro-inflammatory factors to ameliorate the progression of rheumatoid arthritis [37]. In Nrf2 knockout mice, the exacerbation of DA neurodegeneration in SN and increased content of α-synuclein were indicated, which coordinated to aggravate DA neurons loss and neuroinflammation in PD early-stage [38]. Together, Nrf2 activation not only produced anti-oxidative effects, but also regulated redox homoeostasis and attenuated neuroinflammatory responses and thus conferred neuroprotection. Here, this study indicated that ICA inhibited pro-inflammatory factors release during 6-OHDA-induced DA neuronal loss, whereas these inhibitory effects were not demonstrated in Nrf2 knockout mice, implying that the activation of Nrf2 signaling was involved in ICA-mediated anti-neuroinflammatory actions.

Based on the mechanistic studies mentioned above, we suggest that ICA suppressed neuroinflammatory responses and further produced DA neuroprotection through the activation of Nrf2 signaling.

## Conclusion

This study demonstrated that ICA attenuated glia cells-mediated neuroinflammation and evoked DA neuroprotection via an Nrf2-dependent manner. These findings might provide new clues for further investigating the mechanisms underlying Nrf2-mediated neuroprotection and other Nrf2-mediated processes.

## Additional file

Additional file 1: **Figure S1.** ICA protected 6-OHDA-induced DA neuronal damage in WT mice and Nrf2 KO mice. The administration of WT mice and Nrf2 KO mice as described in Figs. 1 and 3. We combined WT and Nrf2 KO mice to detect the protein expression of TH by western blot assay. Results were mean ± SEM from 5 to 6 mice. *p < 0.05 compared with control group in WT mice, ^#^*p* < 0.05 compared with 6-OHDA group in WT mice, ^&^*p* < 0.05 compared with control group in Nrf2 KO mice, ^△^*p* < 0.05 compared with 6-OHDA group in WT mice. (TIF 3082 kb)

## Abbreviations

6-OHDA: 6-hydroxydopamine
AD: Alzheimer’s disease
ALS: Amyotrophic lateral sclerosis
BDNF: Brain-derived neurotrophic factor
DA: Dopamine
DMF: Dimethyl fumarate
GDNF: Glia cells line-derived neurotrophic factor
GFAP: Glia fibrillary acidic protein
HD: Huntington’s disease
HO-1: Heme oxygenase-1
Iba-1: Ionized calcium-binding adapter molecule-1
ICA: Icariin
IL-1β: Interleukin-1β
iNOS: Inducible nitric oxide synthase
Keap1: Kelch-like ECH-associated protein 1
LPS: Lipopolysaccharide
NO: Nitric oxide
NQO1: NADPH quinone oxidoreductase 1
Nrf2 KO: Nrf2 knockout
Nrf2: Nuclear factor erythroid-2 related factor 2
PCNA: Proliferating cell nuclear antigen
PD: Parkinson’s disease
RNS: Reactive nitrogen species
ROS: Reactive oxygen species
SN: Substantia nigra
TH: Tyrosine hydroxylase
TNF-α: Tumor necrosis factor-α
WT: Wild type
