# Supplementary figures and images for: Icariin attenuates neuroinflammation and exerts dopamine neuroprotection via an Nrf2-dependent manner
Source: J Neuroinflammation. 2019 Apr 22;16:92. doi: 10.1186/s12974-019-1472-x (PMC6477740; doi:10.1186/s12974-019-1472-x)

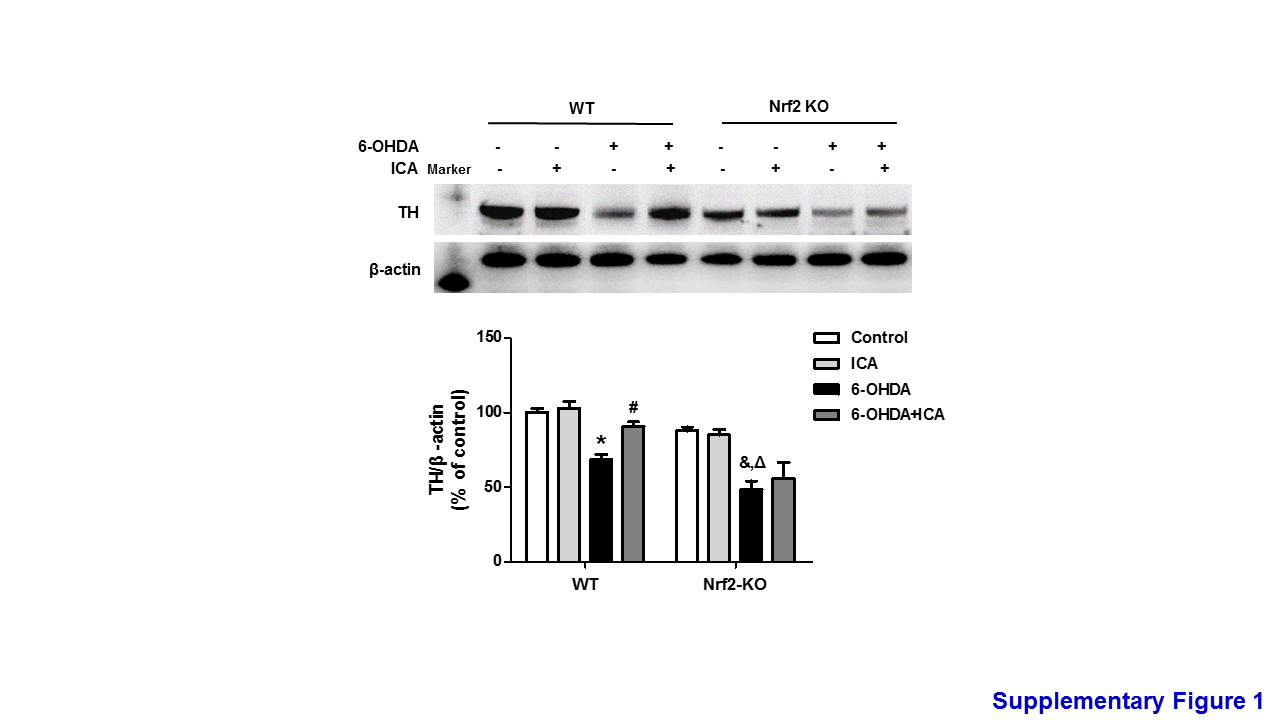

Supplement: Supplementary file 1 — Figure S1. ICA protected 6-OHDA-induced DA neuronal damage in WT mice and Nrf2 KO mice. The administration of WT mice and Nrf2 KO mice as described in Figs. 1 and 3. We combined WT and Nrf2 KO mice to detect the protein expression of TH by western blot assay. Results were mean ± SEM from 5 to 6 mice. *p < 0.05 compared with control group in WT mice, #p < 0.05 compared with 6-OHDA group in WT mice, &p < 0.05 compared with control group in Nrf2 KO mice, △p < 0.05 compared with 6-OHDA group in WT mice. (TIF 3082 kb) [file 12974_2019_1472_MOESM1_ESM.tif]
